# Variation of telomeric repeats in Scots pine (*Pinus sylvestris*) – is there a connection to ageing and loss of regeneration ability?

**DOI:** 10.1186/1753-6561-5-S7-O42

**Published:** 2011-09-13

**Authors:** Tuija Aronen

**Affiliations:** 1 Finnish Forest Research Institute, Punkaharju Unit, FI-58450 Punkaharju, Finland

## 

The shortening of telomeres, the specific structures of repeated DNA sequence at the end of the eukaryotic chromosomes, has been connected with ageing and loss of cell replication or regeneration capacity. There is, however, only limited information available on telomeres of long-living trees. Physiological ageing, on the other hand, represents a serious problem for vegetative propagation of conifers, while in deciduous tree species ageing has no such a strict influence on regeneration ability. The aim of this study was, for the first time, to determine the variation in telomeric repeats in an economically important Nordic conifer, Scots pine (*Pinus sylvestris* L.), and to study potential connections to tree ageing.

Scots pine individuals ranging from immature embryos to 200-year-old trees were examined. In 1- and 5 year-old seedlings and 50-, 100- and 200-year old trees, cambium, bud and needled samples were collected from different positions within a tree. The length of telomeric repeats in the extracted total genomic DNA was then determined by Southern hybridisation. In addition, telomerase enzyme activity was measured from the protein extracts prepared from the same tissues using TRAP (Telomeric Repeat Amplification Protocol) .

In Scots pine samples, the size of telomeric repeats detected was variable, ranging from 0.9 kb up to 26 kb. Based on exonuclease treatment, high molecular weight repeats seem to be genuine telomeres at the ends of the chromosomes, while the low molecular weight signals probably originate at interstitial or centromeric positions. The Southern hybridisation was thus adjusted to show only high-molecular weight signals, and analyses were performed based on them.

A decline in the average length of the telomeric repeats was observed with increasing level of tissue differentiation: immature and germinating embryos had the longest repeats, followed by cambium, elongating buds with tiny meristems and, finally, full-sized needles without meristems having the shortest repeats. When embryo samples were excluded from the data, the age of the tree had no significant effect on the length of telomeric repeats, although there was a tendency for telomere shortening with increasing age. In cambium samples, also the position of the tissue within tree (stem base versus top) affected the length of telomeric repeats, the telomeres shortening towards top of tree. The telomeric repeats also varied remarkably between tree individuals. Potential connections to regeneration ability are discussed.

